# 10 Kernaussagen zur S2k-Leitlinie „Nichtinvasive Beatmung als Therapie der akuten respiratorischen Insuffizienz“

**DOI:** 10.1007/s00063-023-01017-8

**Published:** 2023-05-31

**Authors:** Michael Westhoff, Peter Neumann, Jens Geiseler, Johannes Bickenbach, Stefan Kluge

**Affiliations:** 1 Klinik für Pneumologie, Schlaf- und Beatmungsmedizin, Lungenklinik Hemer, Hemer, Deutschland; 2 Universität Witten-Herdecke, Witten, Deutschland; 3 Klinik für Anästhesiologie und Intensivmedizin, Evangelisches Krankenhaus Göttingen-Weende, Göttingen, Deutschland; 4 Medizinische Klinik IV: Pneumologie, Beatmungs- und Schlafmedizin, Klinikum Vest – Marl, Marl, Deutschland; 5 Klinik für operative Intensivmedizin und Intermediate Care, Uniklinik RWTH Aachen, Aachen, Deutschland; 6 Klinik für Intensivmedizin, Universitätsklinikum Hamburg-Eppendorf, Martinistr. 52, 20246 Hamburg, Deutschland

**Keywords:** Nichtinvasive Beatmung, Akute respiratorische Insuffizienz, Akut exazerbierte COPD, Respiratorentwöhnung, Kardiogenes Lungenödem, Noninvasive mechanical ventilation, Acute respiratory failure, Acute exacerbation of COPD, Weaning from mechanical ventilation, Cardiogenic pulmonary edema

Die nichtinvasive Beatmung (NIV) wird seit über 30 Jahren in der Akutmedizin eingesetzt. Mit dem Einsatz der NIV als Therapieform der akuten respiratorischen Insuffizienz (ARI) werden vor allem bei der hyperkapnischen, zu geringeren Anteilen auch bei der hypoxämischen Insuffizienz die Reduktion der Intubationsrate, der Mortalitätsrate, der Aufenthaltsdauer auf der Intensivstation sowie im Krankenhaus und der Behandlungskosten erwartet. Bei gegebener Indikation sollte die NIV daher als Therapie der akuten respiratorischen Insuffizienz eingesetzt werden, um Komplikationen einer invasiven Beatmung zu vermeiden. Die Leitlinie (AWMF-Register-Nr. 020-004) hat im Vergleich zur vorhergehenden Leitlinie aus dem Jahr 2015 eine umfassende Überarbeitung erfahren und wurde im März 2023 auf der Homepage der AWMF publiziert [1], die Langfassung kann unter diesem Link eingesehen werden: https://register.awmf.org/de/leitlinien/detail/020-004.

Im Folgenden werden die 10 wichtigen Kernaussagen dieser Leitlinie bezogen auf erwachsene kritisch kranke Patienten dargelegt [1]:

## Kernaussage 1

Liegt bei ARI eine der folgenden absoluten Kontraindikationen für eine NIV vor, soll keine NIV eingeleitet werden: 1. fehlende Spontanatmung, Schnappatmung; 2. fixierte oder funktionelle Verlegung der Atemwege; 3. gastrointestinale Blutung oder Ileus; 4. nichthyperkapnisches Koma.

Es bestehen absolute und relative Kontraindikationen für die Durchführung der NIV (Tab. 1). Relative Kontraindikationen bedeuten, dass die NIV im Einzelfall, abhängig von der Erfahrung des Behandlungsteams und der verfügbaren technischen Ausstattung und unter ständiger Intubationsbereitschaft, auch in kritischen Situationen eingesetzt werden kann, dies betrifft insbesondere das hyperkapnische Koma.

**Table 1:** 

Absolute Kontraindikationen	Relative Kontraindikationen
Fehlende Spontanatmung, Schnappatmung	Hyperkapnisch bedingtes Koma
Fixierte oder funktionelle Verlegung der Atemwege	Massive Agitation
Gastrointestinale Blutung oder Ileus	Massiver Sekretverhalt trotz Bronchoskopie bzw. nichtinvasivem Sekretmanagement
Nichthyperkapnisch bedingtes Koma	Schwergradige Hypoxämie oder Acidose (pH-Wert < 7,1)
	Hämodynamische Instabilität (kardiogener Schock, Myokardinfarkt)
	Anatomische u./o. subjektive Interface-Inkompatibilität
	Z. n. oberer gastrointestinaler OP

## Kernaussage 2

Bei einer akuten hyperkapnischen respiratorischen Insuffizienz mit einem pH-Wert < 7,35 soll bei fehlenden Kontraindikationen die NIV eingesetzt werden.

Studien zur Wirksamkeit der NIV bei akut exazerbierter COPD gibt es nunmehr seit > 30 Jahren. Die NIV geht bei dieser Indikation mit einer Reduktion der Intubationshäufigkeit, der Sterblichkeit und der Krankenhausaufenthaltsdauer einher [2]. Bei leichtgradiger akut exazerbierter COPD mit einem pH-Wert ≥ 7,35 besteht dahingegen keine Indikation für eine akute NIV. Neben den Blutgasen und ggf. der transkutanen CO_2_-Messung sind Dyspnoeempfindung und Atemfrequenz die klinisch relevanten Verlaufsparameter zur Beurteilung der Beatmungsqualität in der Adaptationsphase (Tab. 2). Anhand dieser lässt sich bereits 1–2 h nach Beginn der NIV zwischen Respondern (Abnahme dieser Parameter) bzw. Nonrespondern (ausbleibende Abnahme bzw. Zunahme dieser Parameter) unterscheiden. Insbesondere der Anstieg des pH-Werts innerhalb der ersten 4 h ist ein Marker für den Erfolg der NIV. In NIV-Pausen ist der Einsatz der High-flow-Sauerstoff-Therapie bei besserem Komfort und günstigerem Einfluss auf Dyspnoe und Atemfrequenz möglich, die High-flow-Sauerstoff-Therapie sollte jedoch nicht als Primärtherapie eingesetzt werden.

**Table 2:** 

Kriterium	Erfolgskriterien der NIV
Dyspnoe	Abnahme
Vigilanz	Zunehmende Verbesserung
Atemfrequenz	Abnahme der erhöhten Atemfrequenz
Ventilation	p_a_CO_2_-Abnahme
pH-Wert	Anstieg
Oxygenierung	Zunahme von S_a_O_2_ ≥ 85 %
Herzfrequenz	Abnahme

## Kernaussage 3

Ein NIV-Versuch bei komatösen Patienten mit hyperkapnischer ARI ist im Einzelfall gerechtfertigt; er soll allerdings kurzfristig zu einer Besserung der Ventilation und zu einer Vigilanzsteigerung führen; ansonsten soll der Patient intubiert werden.

Bei dieser Patientengruppe ist das engmaschige Monitoring besonders relevant. Bei fehlender Besserung wird eine rasche Möglichkeit der Intubation und eine invasive Beatmung gefordert. Formal gibt es keine untere Grenze für den pH-Wert, unter der ein NIV-Versuch unangemessen wäre. Allerdings ist das Versagensrisiko der NIV umso größer, je niedriger der pH-Wert ist [3].

## Kernaussage 4

Bei persistierender Hyperkapnie (> 53 mm Hg) 2–4 Wochen nach akut exazerbierter COPD mit passagerer NIV sollte eine außerklinische nichtinvasive Beatmung eingeleitet werden.

Auch die Leitlinie zur außerklinischen Beatmung empfiehlt die Evaluation einer außerklinischen NIV nach beatmungspflichtiger akuter respiratorischer Insuffizienz [4]. So sollte spätestens 4 Wochen nach der Beendigung der NIV eine erneute Blutgasanalyse erfolgen, um im Fall einer Persistenz der Hyperkapnie über eine Wiederaufnahme der NIV zu entscheiden. Für die Postexazerbationsgruppe kommt die aktuelle Cochrane-Analyse zum Ergebnis, dass der p_a_CO_2_ durch die NIV sowohl nach 3 Monaten („adjusted mean difference“ [AMD] −0,40 kPa; 95 %-KI −0,70 bis −0,09) und 12 Monaten (AMD −0,52 kPa; 95 %-KI −0,87 bis −0,18) gesenkt wird [5]. Das wiederaufnahmefreie Überleben war unter NIV verbessert („adjusted hazard ratio“ [AHR] 0,71; 95 %-KI 0,54 bis 0,94).

## Kernaussage 5

Bei Patienten mit hypoxämischer ARI bei kardiogenem Lungenödem soll, neben oronasaler Sauerstoffgabe, frühzeitig eine CPAP-Therapie begonnen werden. Dies gilt auch für die Behandlung von Patienten in der Prähospitalphase oder in der Notaufnahme.

Die pulmonale Stauung infolge einer Linksherzinsuffizienz ist die häufigste Ursache der hypoxämischen akuten respiratorischen Insuffizienz. Im Vergleich zur Standardtherapie mit O_2_-Gabe führte CPAP zu einer Reduktion der Krankenhaussterblichkeit und der Intubationsrate, dabei kam es nicht zu einer signifikanten Änderung der Inzidenz von erneuten Myokardinfarkten [6]. Dabei zeigen CPAP und NIV ähnliche Effekte und sind in dieser Indikation als gleichwertig anzusehen. Belastbare Daten hinsichtlich der Effekte einer High-flow-Sauerstoff-Therapie bei akutem Lungenödem im Hinblick auf die Sterblichkeit gibt es aktuell nicht. Eine primäre Therapie mit High-flow-Sauerstoff-Therapie bei hypoxämischer ARI als Folge eines kardiogenen Lungenödems kann aktuell nicht empfohlen werden.

## Kernaussage 6

Bei akuter hypoxämischer ARI (nichtkardial bedingt) mit nur leicht oder moderat gestörtem Gasaustausch (p_a_O_2_/F_i_O_2_ > 150 mm Hg) konnte bislang kein Vor- oder Nachteil von CPAP/NIV im Vergleich zur High-flow-Sauerstoff-Therapie nachgewiesen werden. Daher kann im Einzelfall an Stelle von CPAP/NIV auch ein Therapieversuch mit High-flow-Sauerstoff-Therapie erwogen werden.

Die Datenlage zur Anwendung von CPAP bzw. NIV bei der hypoxämischen akuten respiratorischen Insuffizienz (außerhalb der Indikation kardiogenes Lungenödem) ist insgesamt schwierig zu werten, da eine Hypoxämie bei einer Vielzahl verschiedener Erkrankungen als gemeinsames Symptom resultieren kann und nur wenige Studien prospektiv CPAP bzw. NIV an homogenen Kollektiven mit der gleichen Grunderkrankung (z. B. ambulant erworbene Pneumonie oder Thoraxtrauma mit Lungenkontusion oder sekundäres ARDS bei Sepsis) untersucht haben. Zusätzlich gibt es Hinweise, dass die Art des NIV-Interface (Helm vs. Gesichtsmaske) einen Einfluss auf den Therapieerfolg haben könnte. Eine aktuelle Cochrane-Analyse vergleicht High-flow-Sauerstoff-Therapie mit NIV oder konventioneller Sauerstoffgabe in einem gemischten Patientengut mit akuter hypoxämischer und/oder hyperkapnischer respiratorischer Insuffizienz [7]. High-flow-Sauerstoff-Therapie führt im Vergleich zur konventionellen Sauerstoffgabe bei diesen Patienten lediglich seltener zur Notwenigkeit einer Therapieeskalation, während andere Outcomeparameter nicht oder nicht sicher beeinflusst werden. Im Vergleich zur NIV konnten für High-flow-Sauerstoff-Therapie keinerlei relevante Outcomeunterschiede nachgewiesen werden. Somit scheinen nach gegenwärtigem Wissensstand CPAP/NIV und High-flow-Sauerstoff-Therapie bei Patienten mit hypoxämischer ARI und mild oder nur moderat gestörtem Gasaustausch (p_a_O_2_/F_i_O_2_ > 150 mm Hg) im Hinblick auf das Patientenoutcome gleichwertig zu sein.

## Kernaussage 7

Bei mittelschwerem bis schwerem ARDS (p_a_O_2_/F_i_O_2_ < 150 mm Hg) kann im Einzelfall ein Therapieversuch mit CPAP/NIV und/oder High-flow-Sauerstoff-Therapie unternommen werden.

Dabei sind das klinische Gesamtbild und vorhandene Komorbiditäten zu berücksichtigen. Bei Patienten mit schwerer Hypoxämie (p_a_O_2_/F_i_O_2_ < 100 mm Hg) liegen klare Daten für einen Nachteil durch die nichtinvasive Beatmung vor mit dem Risiko der verzögerten Intubation und erhöhten Mortalität [8–10]. Neben der Schwere des aktuellen Krankheitsbilds ist das Ausmaß der Oxygenierungsstörung ein wichtiger Prädiktor für das NIV-Versagen.

## Kernaussage 8

Der Einsatz von CPAP/NIV bei ARDS soll ausschließlich unter kontinuierlichem apparativem und klinischem Monitoring und ständiger Intubationsbereitschaft erfolgen.

Eine NIV bei akuter respiratorischer Erkrankung erfordert kontinuierliches Monitoring von Respiration und Hämodynamik. Niederschwellig ist bei instabiler Situation eine arterielle Blutdrucküberwachung indiziert. Steigender Sauerstoffbedarf, progrediente Acidose, Auftreten von Apnoen oder zunehmende Erschöpfung erfordern die rechtzeitige, zügige Intubation.

## Kernaussage 9

Bei NIV-Versagen soll die NIV umgehend beendet und unverzögert intubiert werden, sofern keine palliative Gesamtsituation vorliegt.

Das Ausbleiben einer Besserung bzw. die Verschlechterung der in Tab. 2 genannten Parameter, wie auch das Eintreten einer Kontraindikation (siehe Tab. 1), gelten als NIV-Versagen und erfordern den Abbruch der NIV und eine unverzügliche Intubation, sofern keine Palliativsituation vorliegt.

## Kernaussage 10

Die NIV sollte zur Präoxygenierung und unmittelbaren Beatmung vor der endotrachealen Intubation bei akut hypoxämischen Patienten eingesetzt werden.

Es ergeben sich eindeutige Hinweise, dass die NIV einer konventionellen O_2_-Gabe via Gesichtsmaske mit anschließender Maskenbeatmung und einer High-flow-Sauerstoff-Therapie zur Präoxygenierung im Hinblick auf die Häufigkeit und den Schweregrad der S_a_O_2_-Abfälle sowie die Häufigkeit von Komplikationen während des Intubationsvorgangs überlegen ist und deshalb zur Präoxygenierung und Beatmung vor dem Intubationsvorgang bei akut hypoxämischen Patienten eingesetzt werden sollte [11].
